# HXZQCG: A database of natural diverse components in polyherbal formulation HXZQ identified naringenin chalcone as a ligand of H_2_R

**DOI:** 10.1016/j.chmed.2026.06.003

**Published:** 2026-06-10

**Authors:** Shengjie You, Jing Mo, Guoqing Xu, Zhuangyuan Xie, Wenke Xiao, Guiyang Zhang, Yufei Mao, Qi Shen, Leu Kim Fey, Sanyin Zhang, Wei Chen, Liang Leng, Fanbo Meng

**Affiliations:** a Innovative Institute of Chinese Medicine and Pharmacy, Chengdu University of Traditional Chinese Medicine, Chengdu 611137, China; b Chongqing Taiji Industry (Group) Co., Ltd., Chongqing 401123, China; c College of Pharmacy, Hubei University of Chinese Medicine, Wuhan 430065, China; d Institute of Herbgenomics, Chengdu University of Traditional Chinese Medicine, Chengdu 611137, China; e Institute of Medical Plant Physiology and Ecology, School of Pharmaceutical Sciences, Guangzhou University of Chinese Medicine, Guangzhou 510006, China; f M. Kandiah Faculty of Medicine and Health Sciences, University Tunku Abdul Rahman, Perak 31900, Malaysia

**Keywords:** cell membrane chromatography, database, histamine H_2_ receptor, Huoxiang Zhengqi, natural diverse components, polyherbal formulation

## Abstract

Huoxiang Zhengqi (HXZQ) is a classical polyherbal formulation used to treat gastrointestinal disorders. It exerts multi-targeted therapeutic effects, yet its phytochemical complexity hinders standardization and modern pharmacological development. This challenge is further exacerbated by the lack of comprehensive resources covering its diverse natural components, particularly as most previous research neglected peptides and small RNAs, which possess emerging therapeutic potential. There is an urgent need for novel research models to explore polyherbal formulations such as HXZQ. Here, we present the Huoxiang Zhengqi Component Galaxy (HXZQCG), a dedicated database for the HXZQ. HXZQCG integrates over seven million natural components through artificial intelligence-assisted mining, including 15 811 metabolites, 7 597 212 small peptides, and 152 342 small RNAs. To demonstrate its utility, we applied HXZQCG to screen for ligands of the histamine H_2_ receptor (H_2_R), a well-established and druggable G Protein-Coupled Receptor (GPCR) target widely implicated in gastrointestinal pathologies. Through high-throughput screening of potential ligands for H_2_R based on the HXZQCG, we identified 125 candidates. Candidates identified through virtual screening were validated via cell membrane chromatography, ultimately identifying two putative binding small molecules. Furthermore, our results demonstrated that naringenin chalcone is a ligand of H_2_R, and it can inhibit the downstream accumulation of cyclic adenosine monophosphate (cAMP). In sum, HXZQCG stands as a representative case study for polyherbal formulation research. It provides data-based resources for discovery of active component and elucidation of pharmacological mechanisms, serving as a fundamental data-driven platform to support the secondary development of HXZQ. HXZQCG is available at https://cbcb.cdutcm.edu.cn/HXZQCG/.

## 1 Introduction

Traditional Chinese medicine (TCM), with a well-documented history spanning over 2 500 years, plays a significant role in the maintenance of health for people by restoring organismal homeostasis through holistic systemic regulation (Cheung, 2011). Polyherbal formulation contains a wealth of active ingredients that typically exert beneficial synergistic effects on patients by acting on different biological targets. Due to its “multi-components” and “multi-targets” characteristics, it is used to treat various complex diseases (Zhang, An, You, Chen, & Zhang, 2024). The mechanisms of action and chemical basis of bioactivity for numerous TCM compounds await systematic elucidation.

In past research, to accelerate the analysis of herbal active ingredients and the discovery of mechanisms, there has been an explosive growth in studies of natural compounds and their targets, with researchers establishing multiple databases. In the era of big data, these databases serve as valuable resources for both physics-based calculations and artificial intelligence (AI)-driven mining (Zeng, Li, & Wu, 2024). Examples include the Traditional Chinese Medicine Systems Pharmacology Database and Analysis Platform (TCMSP) (Ru et al., 2014), the Herbal Ingredients’ Targets Platform 2.0 (HIT2.0) (Yan et al., 2022) containing compound − target activity pairs, and other resources such as the High − throughput Experiment and Reference − guided Database of TCM (HERB) (Fang et al., 2021), Symptom Mapping (SymMap) (Wu et al., 2018) and the Platform for TCM Knowledge Discovery (TCMKD) (Xiao et al., 2025). These databases have significantly expanded the diversity of secondary metabolites and compound-target pairs in herbal medicines. The recently established Gene-encoded Natural Diversity Components Repository (GNDC) integrates genomic, compositional, and functional data to offer an unprecedented depth of chemical understanding, surpassing the capabilities of conventional natural product databases (Chen et al., 2025). This repository leverages a combination of proprietary and externally developed AI algorithms, alongside customized computational pipelines, for the extraction of components from genomic sequences. Traditional classification of natural products has predominantly focused on secondary metabolites. However, peptides have gained substantial interest in drug discovery due to their low toxicity and high target selectivity. Currently, approximately 80 peptide drugs are commercially available globally, with over 400 candidates in preclinical studies, indicating a progressively expanding pharmaceutical market for peptide-based agents (Muttenthaler, King, Adams, & Alewood, 2021). These peptides regulate physiological processes by targeting diverse protein classes, including ion channels, enzymes, GPCRs, and transporters (Muratspahić, Freissmuth, & Gruber, 2019). Despite extensive characterization of peptides from bacterial, animal, and fungal sources, research on plant-originating bioactive peptides lags significantly, constraining their therapeutic applications. Genomic information-based approaches integrated with artificial intelligence have substantially accelerated the discovery efficiency of plant-derived peptides, yet no specialized resource exists for polyherbal formulations like HXZQ.

Huoxiang Zhengqi (HXZQ) San is a renowned polyherbal formulation since the Song Dynasty of China for treating gastrointestinal diseases. The modern preparation HXZQ, derived from this classical polyherbal formulation, comprises ten medicinal herbs: *Pogostemon cablin* (Blanco) Benth. (Guanghuoxiang, GHX), *Angelica dahurica* (Fisch. ex Hoffm.) Benth. et Hook. f. (Baizhi, BZ), *Magnolia officinalis* Rehd. et Wils. (Houpo, HP), *Pinellia ternata* (Thunb.) Breit. (Banxia, BX), *Perilla frutescens* (L.) Britt. (Zisu, ZS), *Atractylodes lancea* (Thunb.) DC. (Cangzhu, CZ), *Citrus reticulata* Blanco (Chenpi, CP), *Poria cocos* (Schw.) Wolf (Fuling, FL), *Glycyrrhiza uralensis* Fisch. (Gancao, GC), *Areca catechu* L. (Dafupi, DFP). Current pharmacological studies have revealed mechanistic insights into this polyherbal formulation's therapeutic effects, including anti-inflammatory actions (Gao et al., 2022), anti-allergic properties (Sun et al., 2021), modulation of gut microbiota (Wu et al., 2024), and reinforcement of the intestinal mucosal mechanical barrier (Liu et al., 2014). Notably, HXZQ is now included in China's COVID-19 treatment guidelines for GI symptom management, with preliminary studies indicating anti-inflammatory-mediated symptom relief (Tong, Wu, Ni, Shen, & Liu, 2020). Network pharmacology analyses have previously decoded the combinatorial principles and therapeutic mechanisms underlying HXZQ’s efficacy against functional dyspepsia (FD) (Zhao et al., 2019). These studies demonstrate that research on the development and exploration of this polyherbal formulation continues to expand. Further systematic development of classical polyherbal formulations with well-documented efficacy and long-term clinical validation is imperative to unlock their full pharmacological and economic potential. The secondary development of such polyherbal formulations primarily begins with bioactive compound discovery. However, the chemical complexity of HXZQ, particularly its diverse volatile and non-volatile bioactive constituents, necessitates the establishment of a specialized, targeted HXZQ database to facilitate this development process.

This study developed the Huoxiang Zhengqi Component Galaxy (HXZQCG), a specialized database dedicated to HXZQ polyherbal formulation, to systematically update and expand its bioactive constituents. Building upon the methodology of GNDC (Chen et al., 2025), we significantly enhanced formulation’s component coverage. The database now incorporates not only secondary metabolites but also peptides and nucleic acids, representing a major taxonomic expansion. Quantitatively, the annotated secondary metabolites increased 4-fold, while the total data volume expanded by three orders of magnitude Critically, all curated bioactive components and their functional annotations have been integrated into the publicly accessible HXZQCG web platform. Currently, HXZQCG contains over seven million natural components integrated by artificial intelligence, specifically including 15 811 metabolites, 7 597 212 small peptides, and 152 342 small RNAs. This database paves the way for further investigation into the various components of the HXZQ polyherbal formulation, consequently accelerating its secondary development. Furthermore, HXZQCG exemplifies how to construct specialized repositories for other classical herbal formulations.

## 2 Materials and methods

### 2.1 Data annotation and computational processing

The HXZQCG database was constructed through extension and refinement of the framework provided by GNDC (Chen et al., 2025). To systematically profile the HXZQ formulation, a multi-layered data integration and quality control pipeline was established for its three major component classes: metabolites, small peptides, and small RNAs. As a core component of quality assurance, a transparent evidence-level annotation system was implemented. For metabolites, each entry is classified into one of two tiers: Experimentally verified (E-level), confirmed by our in-house liquid chromatography-mass spectrometry (LC-MS) / gas chromatography-mass spectrometry (GC–MS) analyses or database-curated (D-level), sourced from published data within GNDC. For small peptides and small RNAs, entries are uniformly annotated as genome-informed prediction (G-level), reflecting their origin from the genome mining pipeline. All predictive entries underwent standardized filtering and scoring protocols to ensure reliability and traceability.

The metabolite dataset was integrated from GNDC and supplemented with our experimental profiling. For all 15 811 compounds, systematic computational annotation was performed. hierarchical chemical classification (pathways, superclasses, and classes) was assigned using NPClassifier (Kim et al., 2021). Absorption, distribution, metabolism, excretion, toxicity (ADMET) properties were predicted using the ADMET-AI software (Swanson et al., 2024) under default parameters. Drug-likeness scores were generated using a Bayesian Neural Network model (Beker, Wołos, Szymkuć, & Grzybowski, 2020). For the targeted histamine H_2_ receptor (H_2_R) case study, binding affinities were estimated using the graph-sequence fusion framework for drug-target affinity (GSF-DTA) predictor (Zhang et al., 2025), and molecular docking was conducted with AutoDock Vina (v1.2.5) (Eberhardt, Santos-Martins, Tillack, & Forli, 2021), using a grid box centered on the histamine binding site of the H_2_R crystal structure (PDB: 8YN3). Synthetic accessibility (SA) scores were calculated using RDKit (v2023.03.1).

A computational pipeline was established to predict small peptides from the genomic sequences of the ten constituent herbs. Open reading frames (ORFs) were first predicted using orfipy (v0.0.4) (Singh & Wurtele, 2021) with the following parameters: minimum and maximum ORF lengths of 15 bp and 1 000 000 bp, respectively, a block size of 100 for analysis, and recognition of four start codon types (ATG, TTG, CTG, GTG). This process generated a BED12-format ORF annotation file and a corresponding peptide sequence file. The peptide sequences were then filtered using a custom Python script to retain only those initiating with an ATG start codon and with lengths between five and 75 amino acids. Finally, sequence redundancy was reduced using cd-hit (v4.8.1) (Li & Godzik, 2006) with a similarity threshold of 0.5, an alignment word length of 3, a minimum alignment coverage of four amino acids, eight processing threads (−T 8), and a memory limit of 20 000 MB. The output was a non-redundant peptide sequence file for downstream analysis.

The identification of small RNA components involved a structured bioinformatics workflow. To annotate tRNA genes, tRNAscan-SE (v2.0.12) (Chan, Lin, Mak, & Lowe, 2021) was run on each genome in eukaryotic mode (−E), producing separate files for tRNA annotation and secondary structure information. For the systematic identification of other non-coding RNAs (ncRNAs), the cmscan tool (integrated with Infernal v1.1.4) (Nawrocki, Kolbe, & Eddy, 2009) was employed via a custom Python script (v3.10.14). The analysis utilized covariance models from the Rfam database (v14.10) (Kalvari et al., 2021), applied the cut_ga scoring threshold for family-specific inclusion, and specified the corresponding species prefix. The resulting tblout-format file was subsequently converted into a standard GFF annotation file using another Python script, which integrated the annotation details and assigned appropriate species identifiers, yielding a comprehensive small RNA annotation set for each herb.

### 2.2 LC-MS and GC-MS analysis

The following eight medicinal herbs (BZ, HP, BX, CZ, CP, FL, GC, DFP), along with Patchouli oil, Perilla leaf oil, and HXZQ Oral Liquid (Batch number: 22121217) were provided by Taiji Group Chongqing Fuling Pharmaceutical Factory Co., Ltd. (Chongqing, China). All standards used in this study were purchased from Shanghai Yuanye Bio-Technology Co., Ltd. (Shanghai, China). Overall, widely targeted metabolomics analysis was performed on the eight medicinal herbs (BZ, HP, BX, CZ, CP, FL, GC, DFP). GC-MS analysis was performed on Patchouli oil, Perilla leaf oil, and the eight medicinal herbs (BZ, HP, BX, CZ, CP, FL, GC, DFP). Qualitative and quantitative analysis of naringenin chalcone, isoliquiritigenin, and HXZQ Oral Liquid was performed using LC-MS. All these data have been integrated into the HXZQCG database.

The herbal decoction pieces were vacuum freeze-dried in a lyophilizer (Scientz-100F, Zhejiang, China) for 63 h. Subsequently, they were ground into powder using a grinder (MM 400, Retsch, Germany) at 30 Hz for 90 s. Then, 50 mg of the freeze-dried sample powder was weighed, mixed with 1.2 mL of 70% methanolic aqueous (methanol from Merck, Germany), vortexed to mix, and extracted by ultrasonication (KQ5200E, Kunshan, China) for 3 h. During the extraction, the sample was vortexed once every 30 min, for a total of six times. After that, the mixture was centrifuged at 4 °C and 12 000 r/min for 10 min, and the supernatant was collected. The supernatant was filtered through a microporous membrane (0.22 μm pore size, Merck, Germany) and transferred into sample vials for subsequent analysis.

The remaining widely targeted metabolomics analyses were performed by Wuhan Maiwei Biotechnology Co., Ltd. (Wuhan, China). Data acquisition primarily involved ultra-performance liquid chromatography (SCIEX) and tandem mass spectrometry (SCIEX). A C_18_ column (100 mm × 2.1 mm, 1.8 µm) from Agilent was used. The mobile phase consisted of ultrapure water (with 0.1% formic acid) as phase A and acetonitrile (with 0.1% formic acid) as phase B. The gradient elution program was set with 0 min at 5% B, then a linear increase to 95% B over 9 min, held at 95% B for 1 min, followed by a decrease to 5% B from 10 to 11 min, and finally equilibrated with 5% B until 14 min. The flow rate was 0.35 mL/min, the column temperature was 40 °C, and the injection volume was 2 μL. The ESI source operating parameters included a source temperature of 500 °C, an ion spray voltage (IS) of 5 500  V in positive ion mode and −4 500  V in negative ion mode, ion source gas I (GSI), gas II (GSII), and curtain gas (CUR) set to 50, 60, and 25 psi (344.74, 413.69, and 172.37  kPa), respectively, and collision-activated dissociation (CAD) set to high. QQQ scans were performed as MRM experiments with collision gas (nitrogen) set to medium.

GC-MS analysis was carried out using an Agilent Model 8890 GC coupled with a 7000D mass spectrometer (Agilent, USA). First, the herbal decoction pieces were immediately frozen in liquid nitrogen. After complete freezing, they were ground into powder using liquid nitrogen. Then, 500 mg of the powder (or 1 mL of liquid sample) was immediately transferred into a 20 mL headspace vial (Agilent, Palo Alto, CA, USA) containing NaCl saturated solution to inhibit enzymatic reactions. The vial was sealed with a crimp-top caps with a TFE-silicone headspace septum (Agilent, USA). For SPME analysis, each vial was placed at 60 °C for 5 min, and then a 120  µm DVB/CWR/PDMS fiber (Agilent, USA) was exposed to the sample headspace at 60 °C for 15 min.

Separation was achieved on a DB-5MS capillary column (30 m × 0.25 mm × 0.25 μm). Helium (≥ 99.999%) was used as the carrier gas at a linear velocity of 1.2 mL/min. The injector temperature was set at 250 °C, and splitless injection was employed. The oven temperature was programmed as follows: initial 40 °C held for 3.5 min, then increased at 10 °C/min to 100 °C, then at 7 °C/min to 180 °C, then at 25 °C/min to 280 °C, and finally held at 280 °C for 5 min. MS was recorded in electron impact (EI) ionization mode at 70 eV. The quadrupole mass detector, ion source and transfer line temperatures were set, respectively, at 150, 230 and 280 °C. Selected ion monitoring (SIM) mode was used for the identification and quantification of the analytes.

Naringenin chalcone, isoliquiritigenin, and Huoxiang Zhengqi Oral Liquid were analyzed using an ultra-performance liquid chromatography-tandem mass spectrometry (UPLC-MS/MS) system consisting of a Shim-pack UFLC SHIMADZU CBM30A and a SCIEX QTOF 5600 + mass spectrometer. For sample preparation, 1 mL of the original Huoxiang Zhengqi Oral Liquid finished product was taken and mixed with 75% methanolic aqueous at a volume ratio of 1:3 to obtain a mixed solution. After vortexing, the mixture was prepared, and 1 mL of this mixture was centrifuged at 12 000 r/min for 3 min. The supernatant was filtered through a 0.22 μm microporous membrane for further analysis. For standard preparation, 1 mg of each standard compound was dissolved in 75% aqueous methanol to prepare a stock solution, which was further diluted with 75% aqueous methanol to a working concentration of 1 µg/mL before injection.

Separation was carried out on a Waters ACQUITY UPLC HSS T3 column (100 mm × 2.1 mm, 1.8 µm). The mobile phase consisted of (A) 0.1% formic acid in water (*v*/*v*) and (B) 95% acetonitrile in water (*v*/*v*). The flow rate was 0.3  mL/min, the column temperature was maintained at 40 °C, and the injection volume was 2 µL. The gradient elution program was set to 0–0.5 min at 5% B, 0.5–13 min with a linear gradient from 5% to 100% B, 13–16 min holding at 100% B, 16–16.1 min with a linear gradient from 100% to 5% B, and 16.1–20 min holding at 5% B. MS parameters were set with the ESI source operated in positive ion mode, curtain gas at 35 psi (approximately 241.32  kPa), ion source gas 1 at 50 psi (approximately 344.74  kPa), ion source gas 2 at 50 psi (approximately 344.74  kPa), spray voltage at 5 500  V, CAD gas at 7 psi (approximately 48.26  kPa), declustering potential at 80  V, collision energy at 40  V, and ion source temperature at 500 °C.

### 2.3 Construct and verified stable HEK293T-H_2_R cell

Stable transgenic cell lines were established via lentiviral transduction, following method consistent with prior study (Qian et al., 2025). Human embryonic kidney 293 T (HEK293T) cells were purchased from the National Collection of Authenticated Cell Cultures (Shanghai, China), and were cultured in a humidified incubator (Haier, Qingdao, China) at 37 °C with 5% CO_2_. The culture medium was Dulbecco’s modified Eagle’s medium (DMEM, Gibco, Carlsbad, CA, USA) supplemented with 10% fetal bovine serum (FBS, Gibco, Carlsbad, CA, USA) and 1% Penicillin–streptomycin (Gibco, Carlsbad, CA, USA). The key plasmids pCDH-3 × Flag-GFP-Puro, psPAX2, and pMD2.G-were obtained from Youbio (Changsha, China). The plasmids were mixed with Lipofectamine^TM^ 3000 transfection reagent (Thermo Fisher Scientific, USA) and then used to transfect HEK293T cells. Ather 48 h, the supernatants were collected and applied to infect cultured HEK293T cells. Subsequently, the medium was replaced with fresh medium containing 2 μg/mL puromycin (Beyotime, Shanghai, China). Following transfection, GFP fluorescence was observed to assess transfection efficiency. Clones exhibiting high transfection efficiency were selected for subsequent experiments and the expression of the H_2_R protein levels was detected by Western blotting.

### 2.4 Application of Flag-Tagged H_2_R/CMC model

Stably overexpressing H_2_R cells were rapidly thawed in a 37 °C water bath, resuspended in complete culture medium, and then cultured in an incubator at 37 °C with 5% CO_2_. The medium was changed the following day, and cells were passaged every two days. During the exponential growth phase, cells were digested with trypsin and washed three times with PBS (pH 7.4). 5 mL Tris-HCl buffer (pH 7.4) was added to the cell suspension, followed by ultrasonication (200 W, 3 s pulse on/2 s pulse off, 8 cycles) to disrupt the cells. Activated silica gel was sourced from our laboratory, and the cell membrane chromatography (CMC) column was prepared as described previously (Fu et al., 2021). Candidate metabolite standards were obtained from Shanghai yuanye Bio-Technology Co., Ltd. (Shanghai, China). Each standard was dissolved in methanol, and a 5 μL aliquot was loaded into the 2D/CMC analyzer for validation.

### 2.5 Molecular docking

The crystal structure of the histamine H_2_ receptor (H_2_R) was retrieved from the Protein Data Bank (PDB ID: 8yn3). Ligand structures (naringenin chalcone and isoliquiritigenin) were downloaded from the PubChem database (CID: 5280960 and 638278, respectively). For docking preparation, AutoDockTools 1.5.6 was used to add hydrogen atoms and Gasteiger charges to the receptor and ligands, and to generate the required pdbqt files. Molecular docking was performed using AutoDock Vina 1.2.5. The search space (grid box) was centered at coordinates (center *x*  = 99, *y* = 112, *z* = 71), with dimensions of 22 Å × 22 Å × 22 Å (1 Å = 0.1 nm) to adequately cover the binding pocket and surrounding key residues of the target protein. Docking parameters were set as follows: num_modes = 9, energy_range = 10 kcal/mol, and exhaustiveness = 30, to balance conformational sampling depth with computational efficiency.

Following docking, the program estimated and ranked the binding free energies of the generated poses using its empirical and knowledge-based scoring function. The pose with the lowest binding free energy was selected as the most plausible binding mode for subsequent analysis. Two-dimensional ligand protein interaction diagrams were generated using Ligplot to illustrate hydrogen bonds, and hydrophobic interactions. Three-dimensional binding modes were visualized using PyMOL (version 2.5).

### 2.6 Surface Plasmon Resonance (SPR) analysis

SPR analysis was performed on a Biacore T200 system (Cytiva) equipped with a CM5 sensor chip (BR100530, Cytiva). C-terminal 10xHis-tagged H_2_R protein was diluted with acetate buffer (pH 4.0) to a concentration of 20 μg/mL and then injected onto the sample channel at a flow rate of 10 μL/min, achieving an immobilization level of 12 600 response units (RU). Naringenin chalcone was prepared in running buffer (phosphate-buffered saline containing 0.05% (*v*/*v*) Tween-20, PBS-T) at six different concentrations ranging from 0.19 to 6.25 μmol/L. The analyte was injected over both the reference and the H2R protein channels at a flow rate of 20 μL/min, with an association phase of 100 s and a dissociation phase of 180 s. Isoliquiritigenin was prepared in PBS-T at seven different concentrations ranging from 0.19 to 12.50 μmol/L. Each concentration was sequentially injected over both channels at a flow rate of 20 μL/min, with an association phase of 100 s and a dissociation phase of 180 s. All binding data were processed and fitted using the Biacore Insight Evaluation Software (Cytiva) based on a 1:1 Langmuir binding model.

### 2.7 Live cell cyclic adenosine monophosphate (cAMP) assay

Intracellular cAMP concentrations in cultured cells were assayed using previously described (DiRaddo et al., 2014) bioluminescence GloSensor-22F plasmid (Promega), with some modification. 3 × 10^4^ cells/well HEK293T cells were plated in white-walled 96-well plates, and cultured in Dulbecco’s Modified Eagle’s Medium (DMEM, Gibco) at 37 °C with 5% CO_2_. After 24 h, each well received complex containing 50 ng of pGloSensor-22F plasmid, 50 ng of pcDNA3.1-HRH2 plasmid, 20 μL Opti-MEM (Gibco), and 0.2 μL Lipofectamine™ 3000 Reagent, 0.2 μL P3000™ Reagent. After a 24 h incubation, cells were washed twice using Minimum Eagle’s Medium (MEM, Sigma Aldrich) buffer with 20 mmol/L HEPES (Sigma Aldrich) and 0.1% BSA (Sangon biotech). 80 μL of *D*-luciferin sodium salt was adding to a final concentration of 2 mmol/L, and then incubated 2 h in the dark. For antagonist activity, naringenin chalcone was pre-incubated with varying concentrations for 10 min, followed addition 40 nmol/L histamine to all wells. And luminescence was measured immediately using SpectraMax iD3 multi-mode microplate reader 45 min after histamine stimulation, with the detection wavelength set to full spectrum. Data were analyzed using the sigmoidal log (inhibitor) versus dose response function built into GraphPad Prism 9.

## 3 Results

### 3.1 Content of HXZQCG based on GNDC

The newly released HXZQCG represents the first natural diversity component database for polyherbal formulations, encompassing over seven million natural diversity components derived from the ten single herbs in its formulation. These naturally diverse components are systematically classified into three major categories: Secondary metabolites, small peptides, and small RNAs. Genomic information serves as the foundation for AI-driven data mining. In the initial phase of database construction, we systematically collected genomic data for all ten single herbs comprising this classical formulation. To ensure methodological reproducibility, we have detailed in the Materials and Methods section the specific mining and prediction algorithms used for each component category (metabolites, small peptides, and small RNAs) in this study. In addition, the software and parameters Specification employed, as well as the specific quality filters and thresholds integrated throughout the pipeline, are comprehensively described therein.

The Fig. 1 displays the ten individual herbs comprising HXZQ polyherbal formulation, along with their corresponding Chinese names and the genome sizes utilized for data mining. The phylogenetic tree on the left depicts the evolutionary relationships among these plant species. All data presented herein have been integrated into the HXZQCG database and are publicly accessible via its online portal. As shown in Fig. 1, the genome sizes of HXZQ formulation components range from 62 Mb to 4.89 Gb. The largest genome belongs to BZ at approximately 4.89 Gb, while the smallest is FL at about 62 Mb. Additionally, Fig. 1 presents the quantified yields of secondary metabolites, small peptides, and small RNAs identified in each species.

**Fig. 1:**
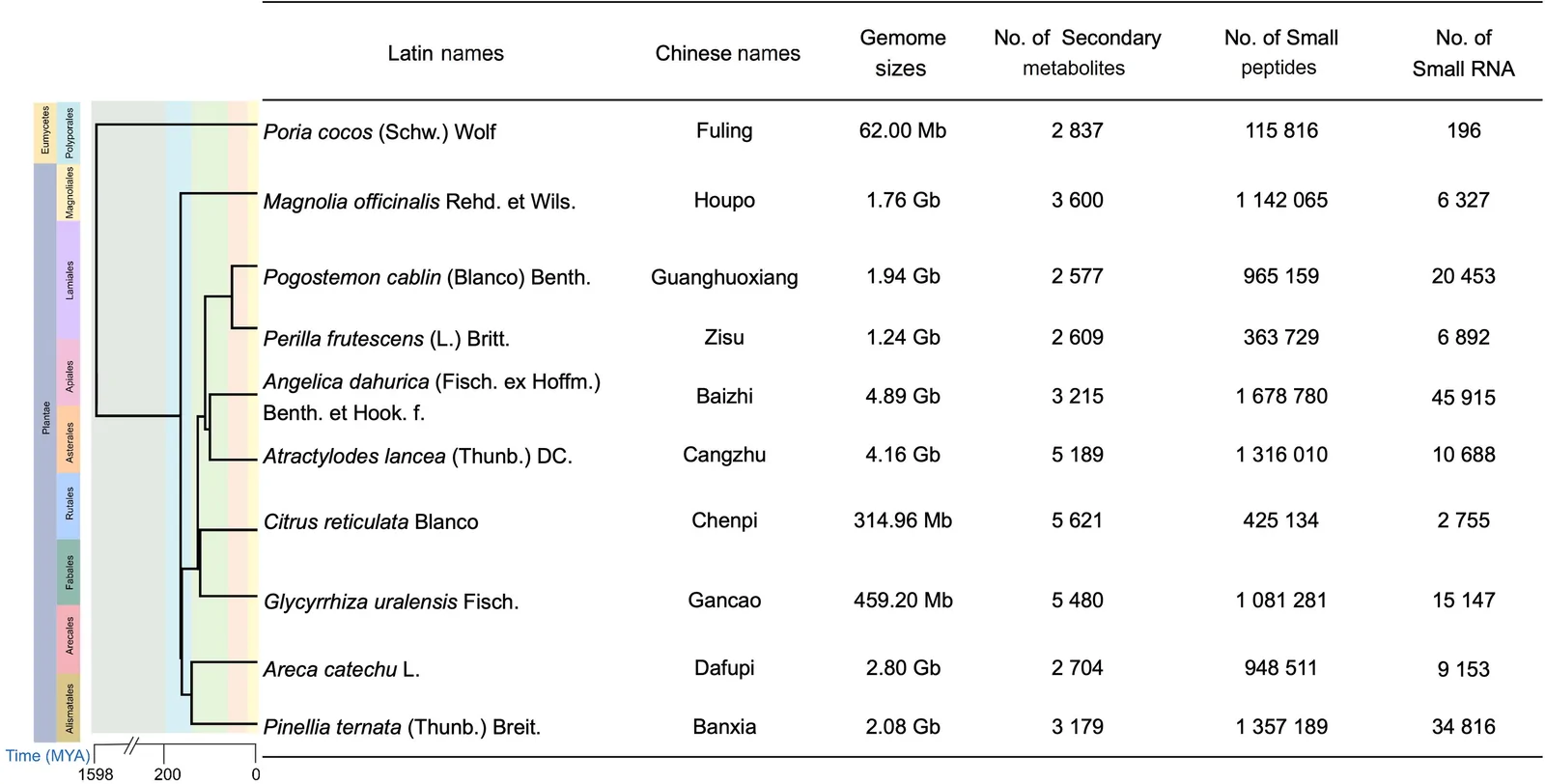
Summarization of diverse components in HXZQ. The ten individual herbs in the HXZQ formulation are shown with their Latin names, Chinese names, genome sizes, and a phylogenetic tree indicating evolutionary relationships. Numbers denote the quantities of secondary metabolites, small peptides, and small RNAs identified.

During the construction of HXZQCG, we identified over 4 000 natural products through LC-MS and GC–MS analyses, and subsequently enriched the dataset with their corresponding SMILES format information. This standardization enabled a cross-referencing comparison with the secondary metabolite repository of GNDC. Previous reports indicated that GNDC encompasses over two million compounds — total of 15 811 compounds associated with the ten herbs in HXZQ were identified through data mining. By employing widely targeted metabolomics and GC-MS analysis, 4 142 metabolites were experimentally detected. This indicates that our experimentally verified data covers approximately 26% of the corresponding entries in GNDC, with a substantial number of remaining compounds awaiting further validation. This strongly suggests that the actual diversity of plant-derived natural products far exceeds currently documented compounds, and their true number is likely much larger than previously estimated. Therefore, the establishment of HXZQCG provides a platform to incorporate these previously unrecorded natural products, and this information will be integrated into GNDC to expand its coverage.

### 3.2 Systematic profiling of secondary metabolites in database

HXZQ is a well-known polyherbal formulation used to treat gastrointestinal diseases (Xiao et al., 2020). Nevertheless, the phytochemical complexity of this polyherbal formulation poses substantial obstacles to standardization and modern drug development. Previous studies have reported secondary metabolites from single herbs in HXZQ such as patchouli alcohol (Wang et al., 2025), perillaldehyde (Zhu et al., 2025), hesperidin and magnolol (Li, Yuan, & Su, 2006). Additionally, researchers have characterized secondary metabolites within the complete HXZQ. Li et al. initially identified 195 components through combined LC-MS and GC–MS analyses (Li et al., 2023). Zhao et al. identified 1 192 chemical constituents in this formulation using structural data retrieved from the NCBI PubChem database (Zhao et al., 2019).

Previous data suffered from fragmentation and lack of standardization, necessitating AI-driven systematic mining and integration to facilitate secondary development of this formulation. Genome-guided mining of the ten single herbs revealed 15 811 secondary metabolites (Fig. 2), systematically categorized into five classes: (i) terpenoids, (ii) shikimate-phenylpropanoids, (iii) fatty acid derivatives, (iv) alkaloids, and (v) other specialized metabolites. Among them, terpenoids accounted for the highest proportion (28.85%), followed sequentially by shikimate-phenylpropanoids (27.22%), fatty acids (7.17%), alkaloids (5.83%), and others (30.93%). We performed in-depth analysis of the mined data by treating the identified secondary metabolites as a discrete dataset. As shown in Fig. 3A, 10 091 (64% of all detected) secondary metabolites were unique to a single species among the ten. Further analysis of these unique compounds revealed that GC contains the highest number of distinct secondary metabolites (2 966), while BX showed no unique metabolites (Fig. 3B). Advances in mass spectrometry and expanding reference libraries now enable detection of trace-level constituents. We performed high-throughput GC–MS analysis of polyherbal formulation, with additional focused characterization of volatile oils (Table S1).

**Fig. 2:**
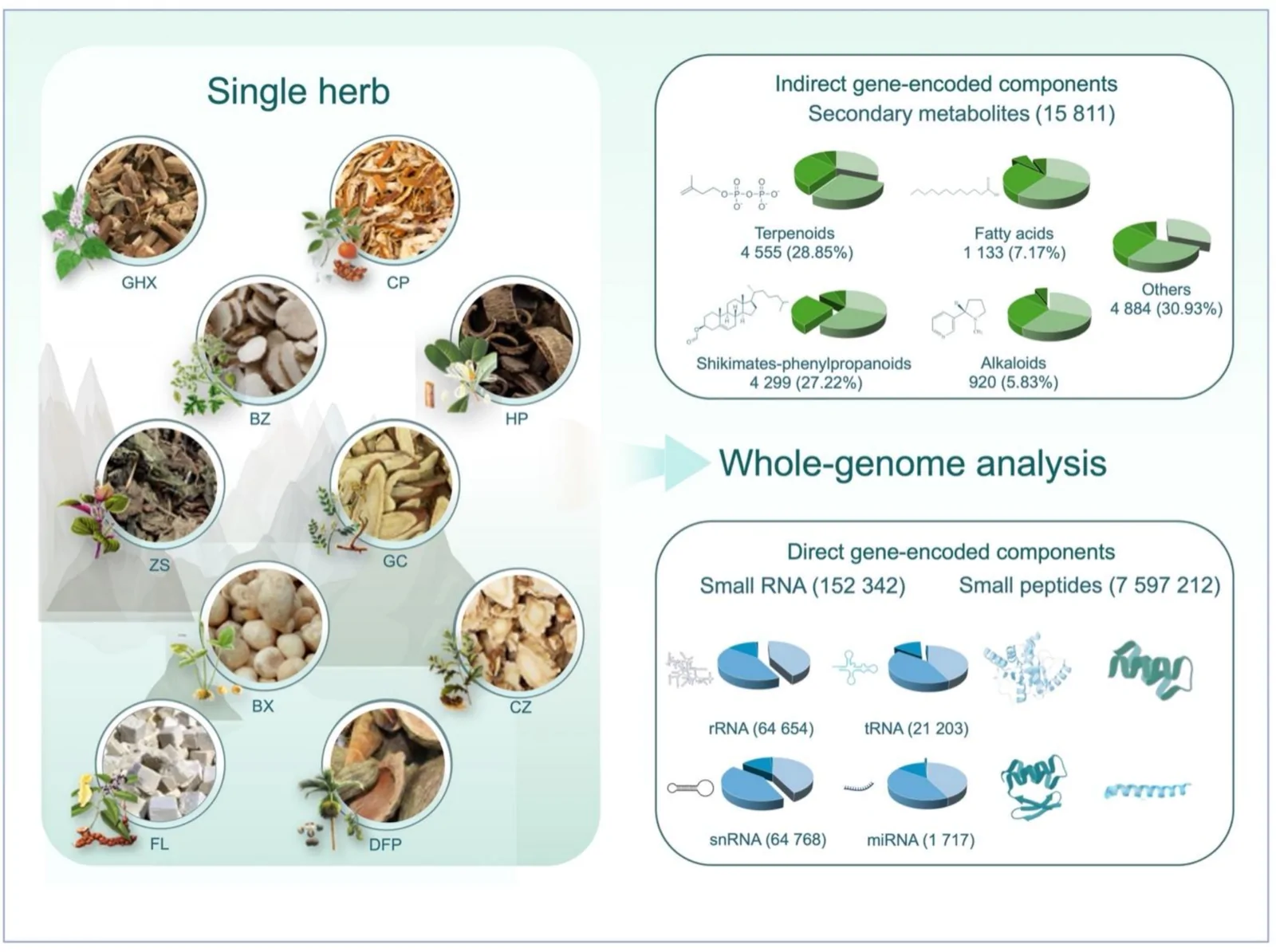
GNDC-based deciphering of direct and indirect gene-encoded components. Following the classification framework of GNDC, natural diverse components are categorized into indirect gene-encoded components and direct gene-encoded components. Indirect gene-encoded components primarily refer to secondary metabolites, and direct gene-encoded components include small RNAs and small peptides. LC-MS and GC–MS analyses were performed on processed herbal slices, while AI‑based mining utilized whole‑plant data. Abbreviations: GHX, Guanghuoxiang; CP, Chenpi; BZ, Baizhi; HP, Houpo; ZS, Zisu; GC, Gancao; BX, Banxia; CZ, Cangzhu; FL, Fuling; DFP, Dafupi; rRNA, ribosomal RNA; tRNA, transfer RNA; snRNA, small nuclear RNA; miRNA, microRNA.

**Fig. 3:**
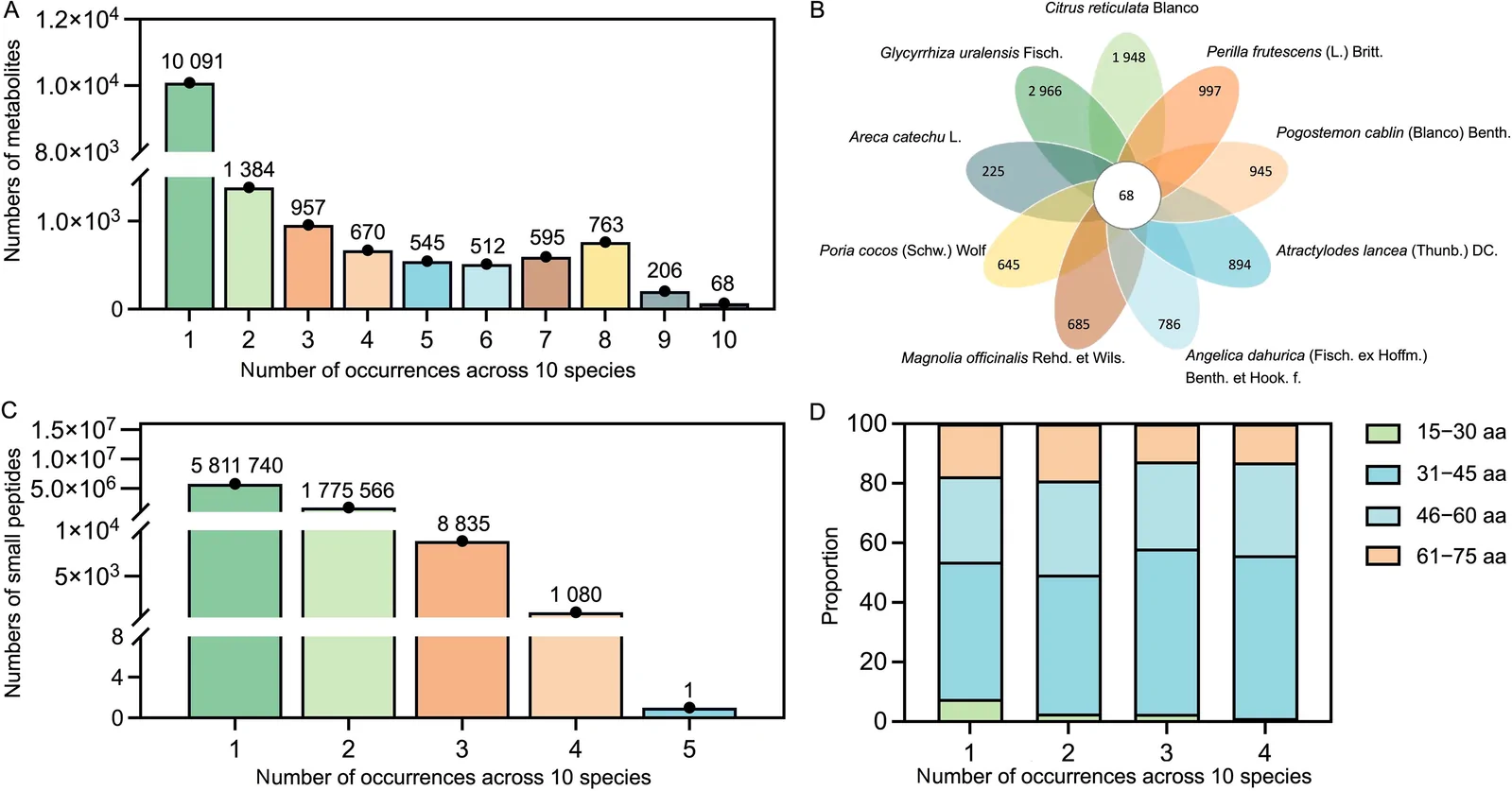
Systematic profiling of secondary metabolites and small peptides in HXZQCG database. **(**A) Metabolites were tallied based on their frequency of occurrence across the ten species. The x-axis represents the frequency of occurrence across the ten species in which a metabolite was found. The numbers atop the histogram indicate the count of metabolites for each frequency. (B). In-depth analysis of the 10 091 metabolites appearing only once (from Panel A). (C). Peptides were counted according to their frequency of occurrence across the ten species. The x-axis denotes the occurrence frequency. The numbers above the histogram indicate the count of peptides corresponding to each frequency. (D). Classification of mined small peptides by length. The x-axis indicates their frequency of occurrence across the ten species.

### 3.3 Systematic profiling of small peptides in database

In this study, comprehensive small peptides profiling was conducted for all ten HXZQ constituent herbs. Leveraging GNDC, we implemented an integrated strategy combining *de novo* genome-based prediction, transcriptome data analysis, and mass spectrometry validation. Detailed methods are provided in the Materials and Methods section. Within this polyherbal formulation, over seven million small peptides were identified in total. These peptides were classified into four categories based on amino acid chain length: 15−30 amino acids (aa), 31−45 aa, 46−60 aa, and 61−75 aa. Among the ten herbs, a remarkable 5.81 million small peptides were found in only one species (Fig. 3C), indicating that over 76% of identified peptides were species-specific. Peptides in the 31–45 aa range constituted the most abundant group, accounting for the largest proportion (Fig. 3D).

Within this polyherbal formulation, the individual herbs originate from eight distinct orders, with Fuling belonging to the Fungal Kingdom. This divergence at the order level and even higher taxonomic ranks partly accounts for the diversity of constituents among the individual herbs. Our data strongly corroborate this point. From the perspective of small peptide mining results, their diversity appears to exceed even that of secondary metabolites. Our earlier analysis revealed a marked contrast: only 10 091 secondary metabolites were specific to a single species, in contrast to the small peptide dataset, which included 5.81 million peptides found exclusively in one species.

Furthermore, functional characterization of peptides in our dataset was performed through comparative analysis against established protein function and pathway databases. This dataset will enhance our ability to identify potential bioactive peptides. For the provided predictive data, these small peptides collectively represent resources that can be further explored and functionally characterized for this polyherbal formulation.

### 3.4 Case study: Screening and validation of active components from HXZQ based on H_2_R cell membrane chromatography

In the modernization of TCM, two primary research avenues involve identifying the active compounds of herbal formulations and elucidating the mechanisms of their targets. As an advanced approach in computer-aided drug discovery (CADD), AI-assisted drug discovery (AIDD) accelerates the completion of key stages, which not only shortens development timelines but also reduces research risks and costs (Zheng et al., 2026). The emergence of artificial intelligence (AI) technologies has accelerated this modernization process, although AI-assisted drug discovery (AIDD) is heavily reliant on the quality and scope of underlying data (Lv et al., 2023). Consequently, the construction of comprehensive and reliable databases is of paramount importance. In the developed HXZQCG, we present a proof-of-concept application through a specific target screening case. Histamine plays a pivotal role in diverse physiological processes, including allergic responses, gastric acid secretion, and inflammatory reactions, primarily mediated through four G protein-coupled receptor (GPCR) members: histamine H_1_, H_2_, H_3_, and H_4_ receptors. Among these, H_1_ receptor antagonists have been used for decades to treat allergic conditions (Tiligada & Ennis, 2020). The H_2_ receptor (H_2_R), predominantly expressed in gastric parietal cells and vascular smooth muscle cells, is widely targeted to inhibit gastric acid secretion. Therapeutically, H_2_ receptor antagonists, such as cimetidine, are clinically employed in the management of gastrointestinal disorders including duodenal and gastric ulcers. HXZQ polyherbal formula has been used to treat gastrointestinal disorders since the Song Dynasty, despite substantial clinical evidence supporting its efficacy, the molecular mechanisms underlying its effects, and the combinatorial principles of its herbal components remain elusive. Thus, we aim to utilize the established HXZQCG to screen for potential active molecules targeting the histamine H_2_ receptor (H_2_R).

Based on our prior data mining efforts, a total of 15 811 components associated with HXZQ were identified. As shown in the virtual screening schematic diagram in Fig. 4A, the top layer represents the compound library mined from HXZQCG. Through sequential filtering based on drug-likeness, ADMET, molecular docking, binding affinity prediction, and compound synthetic accessibility, the candidate pool was narrowed down from 15 811 to 125 compounds. Ultimately, two potential H_2_R ligands, naringenin chalcone and isoliquiritigenin, were validated. The detailed steps are as follows: Applying comprehensive drug-likeness screening reduced the pool of potential molecules by half, resulting in 6 390 candidates (Beker, Wołos, Szymkuć, & Grzybowski, 2020). was performed using multiple ADMET parameters, including compliance with more than four of Lipinski’s rules, QED ≥ 0.5, log*P* < 3, DILI < 0.8, clintox below the 30th percentile of DrugBank compounds, and HIA above the 50th percentile of DrugBank compounds. Molecular docking and affinity filtering (pKd > 5, ΔG <  − 6 kcal/mol), further narrowed this range from 6 390 to 183 candidates. Finally, synthesis accessibility screening (SA score < 2.5) refined the pool to 125 candidates. The list has been provided as a Table S2. Those compounds that are readily commercially available will be selected for subsequent cell membrane chromatography screening (Table S3). Cell membrane chromatography (CMC) is a screening method based on the capacity of membrane receptors to selectively interact with their ligands *in vivo* (Ma et al., 2021). This approach serves as a crucial first step in navigating the vast chemical space of HXZQ, allowing us to prioritize candidate ligands from millions of components for downstream validation. CMC was an efficient filter to bridge *in silico* predictions with initial in vitro binding assessment, a strategy commonly employed in early-stage ligand discovery. In this study, cell membranes extracted from H_2_R-stably overexpressing cells were immobilized onto activated silica gel to prepare an H_2_R-CMC stationary phase for screening. The chromatography results demonstrated that both naringenin chalcone and isoliquiritigenin were capable of binding to H_2_R (Fig. 4E, 4F).

**Fig. 4:**
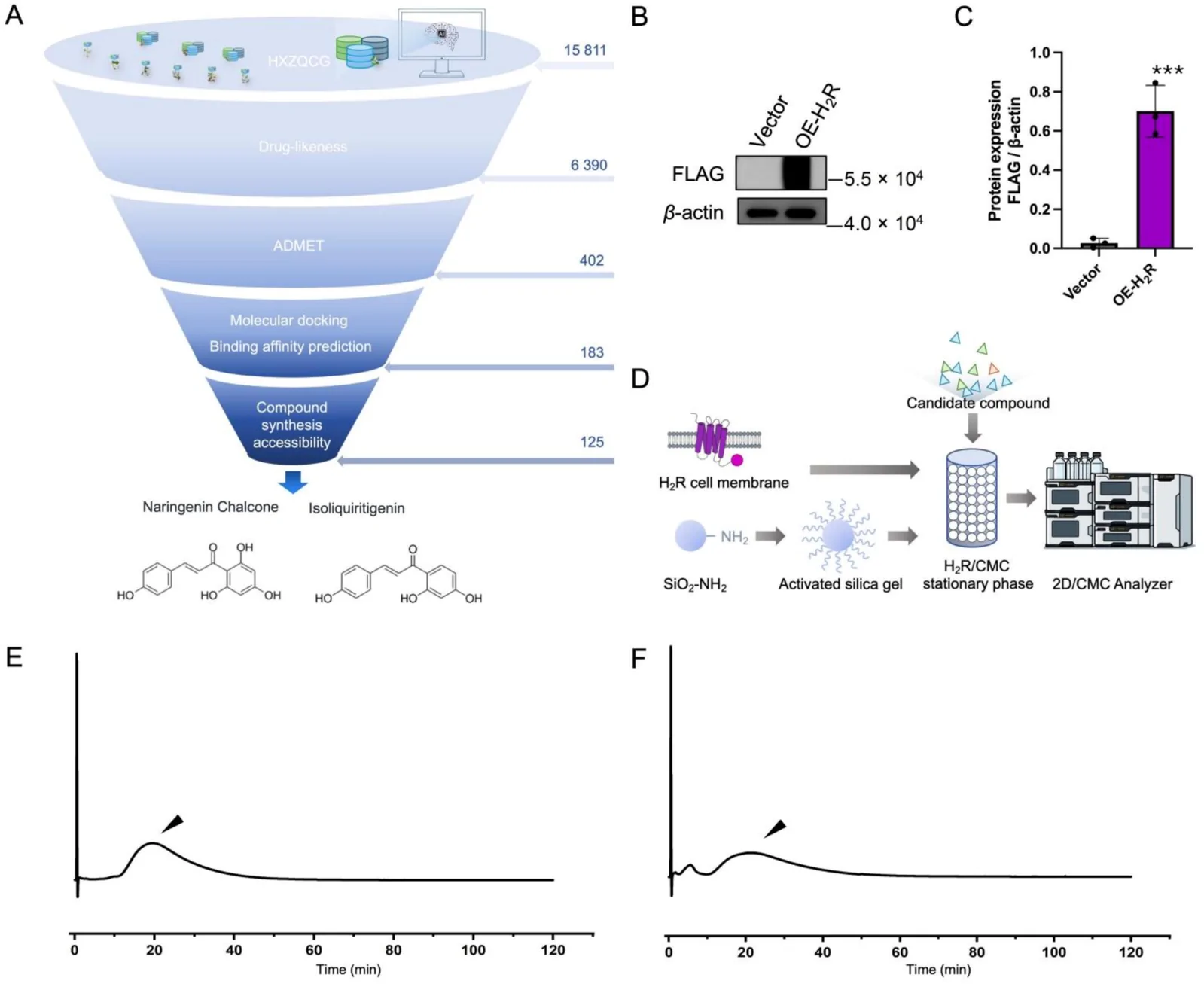
High-throughput screening and validation of active components from HXZQ based on Flag-H_2_R cell membrane chromatography. (A) Schematic of the virtual screening. (B) Protein expression of H_2_R by Western blotting; (C) Relative protein expression levels of Flag-H_2_R in 293 T and OE-H_2_R. Bars represent ± standard deviation (SD) of three independent replicates. Asterisks indicate statistical significance using Student’s test, ^***^P < 0.001. (D) Experimental workflow of the cell membrane chromatography (CMC) assay. (E) Chromatogram of naringenin chalcone on the Flag-tag- H_2_R/CMC. (F). Chromatogram of isoliquiritigenin on the Flag-tag- H_2_R /CMC.

We then analyzed a diluted (10‑fold) commercial sample of HXZQ oral liquid by LC-MS. The red trace represents the compound peaks in the formulation, while the blue trace corresponds to the reference standards. Both naringenin chalcone and isoliquiritigenin were directly detected in the commercial preparation, and their identities were further confirmed by comparing their secondary mass spectra with those of the standards (Fig. 5A−D). It is worth noting that isoliquiritigenin has been previously reported as an effective H_2_R antagonist that does not affect H_1_R or H_3_R signal transduction (Kim et al., 2006). Our screening data are consistent with these literature findings, validating the reliability of the HXZQCG screening approach. Furthermore, molecular docking simulations were performed, which calculated a binding energy of –8.0 kcal/mol for the interaction, as illustrated in the corresponding Fig. 5E−H. SPR results demonstrated that naringenin chalcone and isoliquiritigenin bind to the recombinant H_2_R protein in a concentration‑dependent manner, with binding signals increasing as the compound concentration increased. The calculated KD values were 9.1  μmol/L for naringenin chalcone and 11.1  μmol/L for isoliquiritigenin (Fig. 5I, J). As shown in Fig. 5K, L, histamine induced cAMP increased and naringenin chalcone inhibited cAMP increased in a concentration-dependent manner.

**Fig. 5:**
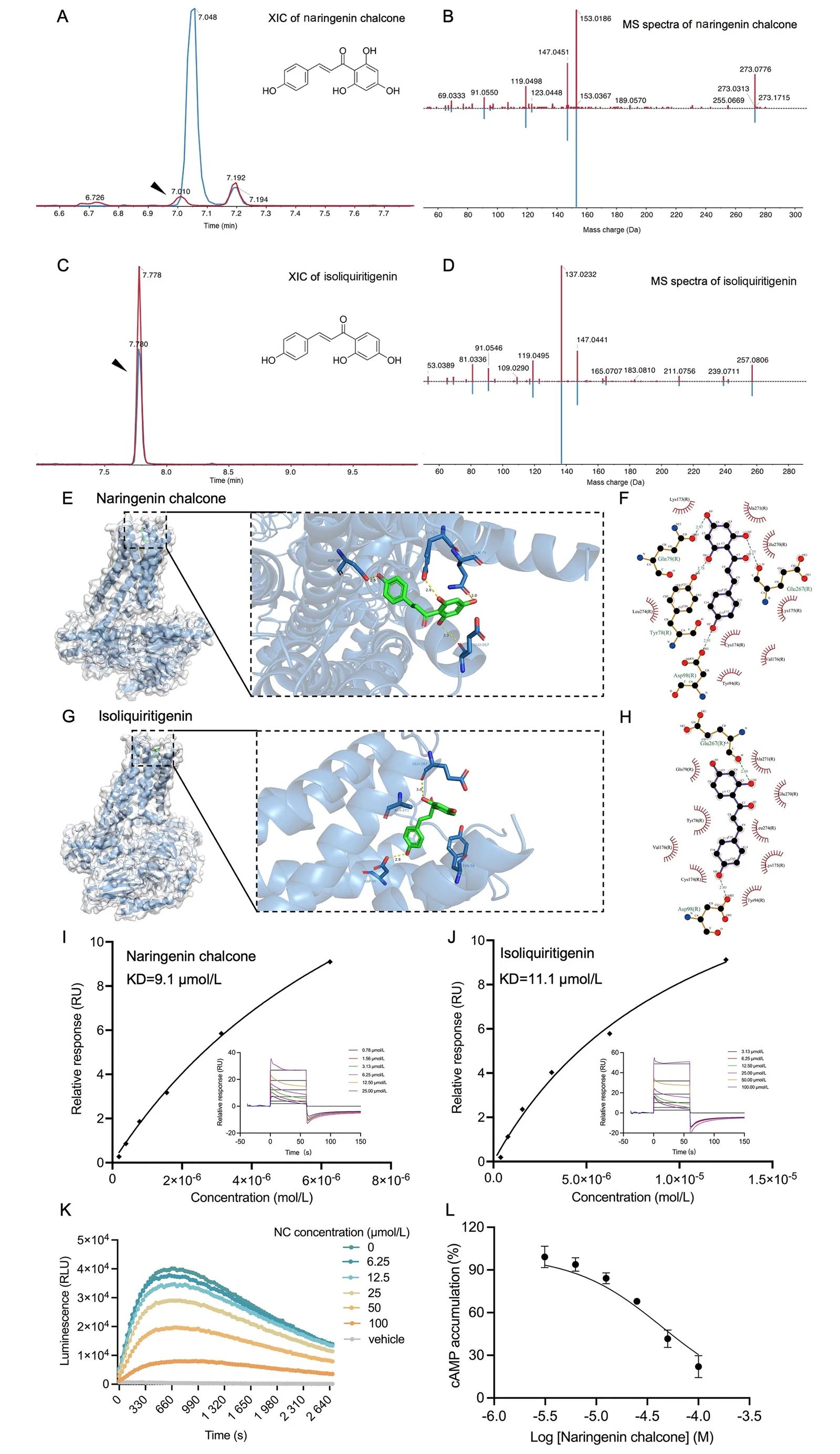
Experimental verification showing that the components in HXZQ are ligands of H_2_R. (A, B) The LC-MS analysis of naringenin chalcone in HXZQ. The blue trace represents the reference standard, and the red trace corresponds to the diluted HXZQ sample. Panel (A) shows the Extracted Ion Chromatogram (EIC) of naringenin chalcone. Panel (B) displays the MSMS spectrum of naringenin chalcone. (C, D) The LC-MS analysis of isoliquiritigenin in HXZQ. (E, F) Molecular docking result and 2D interaction diagram of naringenin chalcone. Green dashed lines represent hydrogen bonds, and red lines indicate hydrophobic interactions. (G, H) Molecular docking result and 2D interaction diagram of isoliquiritigenin. (I, J) SPR sensorgrams and the fitting curves of equilibrium dissociation constant (KD) of naringenin chalcone and isoliquiritigenin. (K, L) GloSensor based cAMP accumulation assays. (K) Kinetic profiles of naringenin chalcone at increasing concentrations on inhibited cAMP accumulation, as measured by GloSensor luminescence in HEK293T cells expressing histamine H_2_ receptor (H_2_R) and the cAMP biosensor pGlosensor-22F; (L) Concentration–response curves of naringenin chalcone-mediated inhibition of cAMP accumulation. Data points represent the means ± SEM from three independent experiments.

### 3.5 Database construction for HXZQ

HXZQCG is a specialized natural component database for the classical polyherbal formulation HXZQ. This resource integrates genomic information from all ten constituent (GHX, BZ, HP, BX, ZS, CZ, CP, FL, GC, DFP) and genomic mining and artificial intelligence to expand natural component coverage. As shown in Fig. 6A, the homepage of the website features botanical images of each herb alongside corresponding photographs of their processed herbal slices. Users can enter keywords in the search box at the top of each component listing page to perform searches. Regarding website usage, we have provided a dedicated “Help” category to assist users. which includes a “Quick Start Guide” (containing a database overview, search functionality, and data display) and examples illustrating the use of the website's analytical tools. Currently, we display the version identifier (v1.0.0) at the bottom of the website. Researchers will be able to track the initial release and any future modifications through a dedicated “Release/Update Log”.

**Fig. 6:**
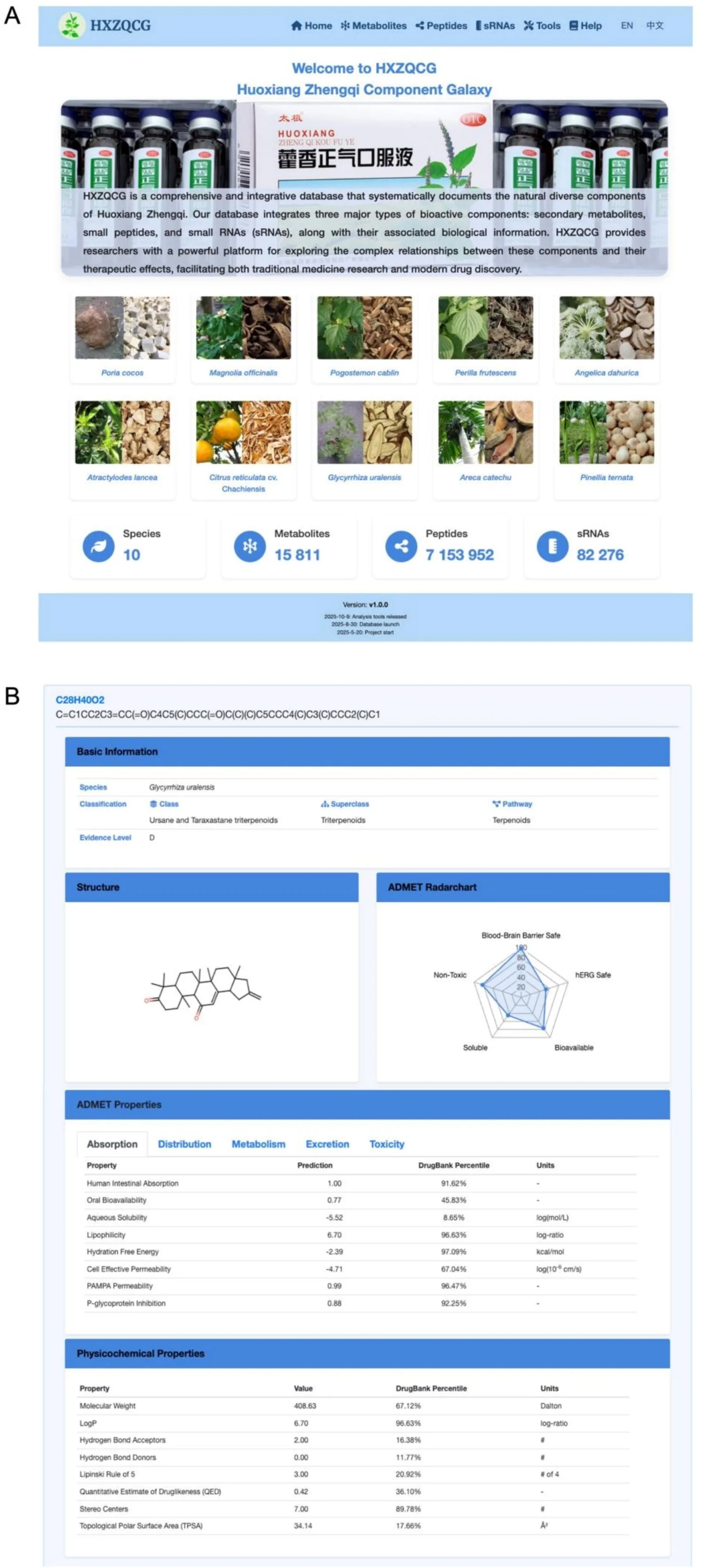
Screen shots of the web interfaces of the data composition and module setting. (A) Screenshot of the HXZQCG homepage. (B) The metabolite search interface. This page comprises five modules: Basic information, Structure, ADMET radar chart, ADMET properties and Physicochemical properties.

The secondary metabolites module catalogs 15 811 compounds. Advances in mass spectrometry and expanding reference libraries now enable detection of trace-level constituents. We performed high-throughput LC-MS/MS analysis of polyherbal formulation, with additional focused characterization of volatile oils. To enhance entry traceability, the detailed page for each database entry now explicitly displays its assigned Evidence Level (E/D), corresponding to: E (Experimentally Verified) and D (Database-curated). By clearly distinguishing entries according to this evidence-based tier, the system provides immediate transparency regarding the foundational support—whether experimental, curated, or predicted. Beyond standard compound information (name, SMILES, species, common names, structure), the platform provides ADMET profiles for pharmacological screening and evaluation (Fig. 6B). On the functional module front, HXZQCG introduces three new analytical tools: Compound Similarity, Peptide Properties, and RNA Structure. The remaining module maintains functional consistency with the established GNDC database (Chen et al., 2025). Critically, HXZQCG is a specialized, curated, and application-oriented database. Rather than a general-purpose repository, it is designed to provide a focused starting point and analytical environment for studying a specific polyherbal formulation (PHF), to facilitate its systematic investigation and ultimately unlock its pharmaceutical potential.

## 4 Discussion

TCM has gradually been widely used around the world for the treatment of complex diseases. HXZQ polyherbal formulation originated from the ancient Chinese pharmacy book “Formularies of the Bureau of the People's Welfare Pharmacies”. A well-known Chinese herbal formula, HXZQ has been used for thousands of years to treat gastrointestinal disorders. The natural ingredients derived from different herbs in this polyherbal formulation provide a variety of pharmacological functions, including anti-inflammatory, gastrointestinal motility regulation, and immune regulation. Presently, the re-development of HXZQ faces the challenge of identifying new clinical applications and target populations, with elucidating the active components and mechanisms of action of TCM being a critical component of this process.

This formula contains ten herbs, each of which often contains hundreds or even thousands of different natural components. Although a series of analytical methods, such as high-performance liquid chromatography (HPLC) and LC-MS/MS, have been developed to determine the components of TCM, it is very difficult to identify all the components in a formula. In fact, methods for detecting Chinese herbal medicine components are also a hot topic in the field of Chinese herbal medicine research. The lack of Chinese herbal medicine components hinders researchers' understanding of their mechanisms of action. The continuous upgrading of AI is revolutionizing drug development methods, and databases are the raw materials for AI research and development. There is an urgent need for a dedicated database to support classic polyherbal formulation with proven clinical efficacy. Within the framework of this particular investigation, the HXZQCG was established and implemented through the application of the GNDC methodology, serving as a representative case study for polyherbal formulation (PHF) research. GNDC helped gather natural diverse components, followed by experiments linking HXZQ's ability to cure a disease with possible components. Based on the resources of the GNDC, this database reorganizes and mines the natural diverse components in this polyherbal formulation and forms an open, accessible website. It should be noted that we did not exhaustively analyze the numerous components of HXZQ to elucidate the precise mechanism by which HXZQ treat the disease. Instead, this investigation presented a cohesive flow chart to illustrate the feasibility of identifying a putative target receptor and its associated ligand involved in disease treatment. Consequently, the HXZQCG and the current work facilitates subsequent research into the other components of the HXZQ polyherbal formulation, provided candidate components and a defined route for their examination. With more integrated and comprehensive data on the natural diverse components of the formula, it will help elucidate the mechanism of action of the formula.

Compared to GNDC, the construction of HXZQCG has enabled a significant expansion and enrichment of its contents. In terms of data foundation, we have clarified that while the overall framework was adapted from GNDC, the HXZQCG construction involved re-running the entire pipeline exclusively on the ten herbs constituting the formulation, applying herb-specific parameters (e.g., genome files, species prefixes). In addition, taking secondary metabolites as an example, through the precise reconstruction of this formulation, we have supplemented additional natural product derived from its ten constituent herbs, with detailed source annotations provided for each. The new three-tiered evidence-level annotation system has been added to HXZQCG, which was not present in the original GNDC. By clearly distinguishing entries based on their underlying evidence (E/D/G) and documenting the quality filters applied, we aim to prevent the overestimation of data reliability and empower users to make informed decisions when utilizing HXZQCG for their research applications. On the functional module front, HXZQCG introduces three new analytical tools: Compound Similarity, Peptide Properties, and RNA Structure. While GNDC serves as a comprehensive platform for natural diversity products in general, HXZQCG establishes a specialized research pathway for in-depth PHFs studies, thereby contributing to the further refinement and utility of GNDC for broader research.

HXZQCG is the first natural ingredient database for classic polyherbal formulation. Compared to previously reported databases, HXZQCG enhances data coverage by integrating more than seven million natural diverse components, which is three orders of magnitude more than the previously reported data. HXZQCG is a specialized, curated, and application-oriented database. It serves a distinct purpose: to provide a focused starting point and analytical environment for studying a specific classical formulation, thereby reducing the preparatory burden for researchers and enhancing the relevance of the data. Although the establishment of HXZQCG has facilitated the research and development of HXZQ formulation, we still face some challenges. For example, the uncertainty and potential bias of data mined by AI. In secondary metabolites section, in addition to covering the actual measured compounds, more than 50% of the compounds require more time for correction. Similarly, in the mining of small peptides, there are currently more than seven million data points. For researchers engaged in mechanism analysis, how to screen and identify small peptides with core functions from a large amount of data is a challenge.

Simultaneously, we used H_2_R as a case study to demonstrate the data analysis of HXZQCG, successfully validating a novel ligand naringenin chalcone. As a naturally occurring flavonoid precursor found in various foods and herbs, naringenin chalcone is generally regarded as safe. While naringenin chalcone shows H_2_R antagonistic activity in our assay, we note that elucidating its underlying mechanisms requires more comprehensive functional validation. This includes determining its Kd values, conducting gastrointestinal cell-based assays, and performing *in vivo* animal studies. The potential of naringenin chalcone resides in its role as a natural multi-target agent within the polyherbal formulation, supporting integrated gastrointestinal modulation, not as a direct drug substitution. However, from the perspective of identifying active components, the validation efficiency via cell membrane chromatography remains limited. Given the multitude of targets associated with gastrointestinal diseases and the extensive component library provided by HXZQCG, we propose that future studies could adopt the Multi-Target Cell Membrane Chromatography Intelligent Screening Workstation (MCMC-ISW). This system integrates “multi-target synchronous recognition” and “intelligent screening” to significantly enhance screening throughput (Zhang, He, & Lu, 2026).

In the future, with the increasing availability of new high-throughput data and the optimization of artificial intelligence, HXZQCG will be regularly updated and corrected. In addition, the development team will continue to expand the collection of natural diversity components and strengthen the functional identification of large amounts of small peptide data. In subsequent iterations of HXZQCG (e.g., Version 2.0.0), following the completion of core dataset development, we plan to expand its scope by incorporating additional dimensions such as clinical data. These new measures are expected to improve the application efficiency of HXZQCG.

## CRediT authorship contribution statement

**Shengjie You:** Conceptualization, Formal analysis, Data curation, Investigation, Writing – original draft, Writing – review & editing, Validation, Visualization, Project administration, Funding acquisition. **Jing Mo:** Writing – original draft, Visualization, Validation. **Guoqing Xu:** Formal analysis, Data curation. **Zhuangyuan Xie:** Validation, Visualization. **Wenke Xiao:** Data curation. **Guiyang Zhang:** Data curation, Visualization. **Yufei Mao:** Data curation, Visualization. **Qi Shen:** Resources. **Leu Kim Fey:** Supervision. **Sanyin Zhang:** Supervision, Conceptualization. **Wei Chen:** Resources, Conceptualization. **Liang Leng:** Supervision, Conceptualization, Formal analysis, Writing – review & editing, Project administration. **Fanbo Meng:** Methodology, Software, Formal analysis, Writing – review & editing, Data curation.

## Declaration of competing interest

The authors declare that they have no known competing financial interests or personal relationships that could have appeared to influence the work reported in this paper.

## Appendix A Supplementary data

Supplementary Data 1 The supplementary data include Figures and three Tables.
