# Supplementary material for: HXZQCG: A database of natural diverse components in polyherbal formulation HXZQ identified naringenin chalcone as a ligand of H2R
Source: Chin Herb Med. 2026 Jun 10;18(4):807–19. doi: 10.1016/j.chmed.2026.06.003 (PMC13712359; doi:10.1016/j.chmed.2026.06.003)
Supplement: Supplementary file 1 [file mmc1.docx]

**Supplementary materials**

**Fig. S1** The full-membrane images for Fig 4B. Expression of H_2_R in the cell line was confirmed by Western blot, as shown in the figure. On the left, anti-FLAG antibody staining shows a strong band in the OE-H_2_R compared to the control. On the right, anti-β-actin antibody results are shown, with all experiments performed in triplicate.

**Fig. S2** Vector map for overexpression of histamine H2 receptor (H_2_R) in HEK-293T cells. In our study, we selected a classic lentiviral expression vector PCDH-GFP as the backbone, which includes a fluorescent reporter gene driven by a separate promoter to facilitate fluorescence‑based screening during cell line establishment. In addition, a 3×FLAG tag was engineered at the C‑terminus of the H2R protein to enable detection of the target protein.

**Fig. S3.** A series of cell membrane chromatograms for the candidate compounds. In the experimental screening, eight candidate compounds were analyzed by cell membrane chromatography (CMC). A CMC column was prepared using the stably H_2_R‑overexpressing cell line, following the protocol detailed in Section 2.4 (“Application of the Flag‑Tagged H2R/CMC Model”) of Materials and Methods. All candidate molecules were purchased from Shanghai Yuanye Bio‑Technology Co., Ltd. Among these, naringenin chalcone and isoliquiritigenin, which yielded positive results, have been characterized in detail within the manuscript. Chromatograms corresponding to hesperidin, glycyrrhizic acid, narirutin, poricoic acid A, magnolol, and honokiol are shown in Panels A–F of Fig. S3. Further details are provided in Supplementary Table 3.

**Fig. S4.** Negative control of the cell membrane chromatography (CMC). (A) Chromatograms of naringenin chalcone on the GFP CMC mod. (B) Chromatograms of Isoliquiritigenin on the GFP CMC mod.
